# Dynamic ultrasound guided assessment of pelvic ring injury measurement (PRIME) for traumatic symphysis diastasis

**DOI:** 10.1007/s00068-025-02973-w

**Published:** 2025-10-28

**Authors:** Till Berk, Felix Karl-Ludwig Klingebiel, Giovanni Colacicco, Beatrice A. Lauber, Dominic Gascho, Yannik Kalbas, Christian T. Hübner, John Ricklin, Frank Hildebrand, Hans-Christoph Pape, Sascha Halvachizadeh

**Affiliations:** 1 Department of Orthopedic Trauma Surgery, RWTH Aachen University, Aachen, Germany; 2 Klinik für Traumatologie, Universitätsspital Zürich, Universität Zürich, Rämistrasse 100, Zürich, 8091 Switzerland; 3 Harald-Tscherne Laboratory for orthopedic and Trauma research, University of Zurich (UZH), Sternwartstrasse 14, Zurich, 8091 Switzerland; 4 Institute of Anatomy, University of Zurich (UZH), Winterthurerstrasse 190, Zurich, CH - 8057 Switzerland; 5 Institute of Forensic Medicine, University of Zurich, Zurich, Switzerland; 6 Department of Trauma, University Hospital Zurich, Raemistrasse 100, Zurich, 8091 Switzerland

**Keywords:** E-FAST-PRIME, Symphysis diastasis, Pelvic trauma, Anterior pelvic ring injury, Trauma bay diagnostics, Primary survey

## Abstract

**Background:**

Assessment of pelvic ring injuries in the pre-clinical and trauma bay setting represents a challenge for the treating trauma team. The objective of the present project was to conduct a pre-clinical trial to investigate the feasibility and accuracy of ultrasound (US) guided assessment of symphyseal diastasis, of cadavers with pelvic ring injuries.

**Methods:**

This is a prospective, anatomical, interventional and radiological cadaveric laboratory investigation. Cadavers were prepared with a pelvic ring injury (symphyseal diastasis). Eleven trauma surgeons performed an ultrasound-guided assessment of the symphyseal diastasis. The intervention was performed in four formalin-fixes cadavers. One served as the control and the other were prepared to have a set of standardized symphysis diastasis. The diastasis was grouped into “below 2.5 cm” and “above 2.5 cm”. Trauma surgeons were blinded to the symphysis diastasis and performed an ultrasound-guided assessment of the anterior pelvic ring. Sensitivity and specificity analyses were performed.

**Results:**

The ultrasound measure of the control provided a sensitivity of 0.73 (95%CI 0.39 TO 0.94). The sensitivity of the ultrasound measure increased with increasing diastasis of the symphysis (Group above 2.5 cm 0.82, 95%CI 0.48 to 0.98). The highest sensitivity was measured in the dynamic assessment of the symphysis closure (0.91, 95%CI 0.59 to 1.0). Specificity was lowest in Group below 2.5 (0.73, 95%CI 0.39 to 0.94).

**Conclusion:**

Ultrasound-guided assessment of symphyseal diastasis is feasible and shows high diagnostic accuracy, especially for diastasis > 2.5 cm and during dynamic evaluation. Sensitivity and predictive values improved with larger displacements, while detection of smaller diastasis remained limited. These findings support the potential use of ultrasound as a rapid, non-invasive tool in early pelvic trauma assessment.

## Introduction

When treating major trauma patients, the rapid detection of life-threatening injuries has urgent priority. Main bleeding sources are besides the chest, the abdomen and the femur, the pelvis [1]. Pelvic ring injuries are results of high-energy trauma in the young population. Especially these injuries are potential lethal and require immediate assessment and accurate diagnostics [2]. In the preclinical setting the assessment of pelvic ring injury bases on the trauma mechanism, patient’s history of pain, and clinical assessment of pelvic ring instability [3, 4]. In the trauma bay setting, the assessment of the pelvic ring injury is an integrated part of the primary survey and is recommended to be assessed in every trauma patient with appropriate history. According to the ATLS® guidelines, the primary survey includes these detections with the support of several adjuncts such as conventional radiographic imaging or ultrasound of several body cavities [1].

Conventional x-ray diagnostics and computed tomography (CT) scans are the gold standard in the detection of bony pelvic injuries and traumatic symphysis ruptures. These are utilized in the evaluation of severely injured trauma patients in the trauma bay. CT diagnostics has the highest sensitivity in the detection of pelvic injuries [5]. However CT-scans in unstable trauma patients are time consuming and where described as ‘doughnut of death’ [5]. Further, in suspected cases of potential pelvic ring injury, a pelvic binder is utilized to reduce and stabilize the potential injury [6]. The application of the pelvic binder requires a certain amount of force that is not standardized and only monitored through the pathophysiologic response of the patient. It highly depends on the experience of the applying individual. Too high or insufficient compression can only be assessed with a considerable amount of experience or due to imaging [7]. If applied correctly, the application of a pelvic binder might mask pelvic ring injuries, such as open book injuries, during the initial radiographic assessment [8]. The release of the pelvic binder might further benefit from algorithms [9].

The aim of the present study was to prove the feasibility and applicability of a new ultrasound (US) guided assessment of symphyseal diastasis in pelvic ring injuries. The hypothesis was tested if the rapidly available, radiation-free dynamic US of the symphysis could be used to establish whether injuries to the symphysis can be diagnosed and whether the correct compression of the pelvic belt can be assessed. To our knowledge, a study of this kind has not yet been conducted and is therefore underrepresented in the current literature.

## Methods

In this prospective, anatomical, interventional and radiological cadaveric laboratory investigation, we included five formaline-fixe cadavers for research purposes in January 2025. Each cadaver received an open book fracture. The symphysis was surgically prepared using a stoppa approach. The symphysis was disrupted. An AO distractor was placed in the disrupted symphysis and the diastasis was performed. One cadaver served as the control. The diastasis was measured with a ruler and held in position with an external fixator that was placed in the usual manner. The skin was closed in all cadavers. The control cadaver also received an incision and the application of an external fixator. At baseline a radiological technologist performed a computer tomographic (CT) scan of the pelvis. The CT scans were performed using a 128-slice CT (SOMATOM Definition Flash, Siemens Healthcare, Forchheim, Germany). A CT scan over the entire pelvis was performed. The scan parameters were 120 kVp, 800 mAs, and a pitch of 0.35. The raw data were reconstructed using a slice thickness of 0.6 with a hard kernel (B60) and a soft kernel (B30). After imaging, we transported the bodies back to the “Skills Laboratory” of the Institute of Anatomy and placed them into supine position.

The next step was the preparation of the pelvic binder. It was applied in the usual manner so that the middle part of the pelvic binder was above the major trochanteric region [10].The pelvic binder was initially not tightened. The symphysis was from the participants assessed utilizing a mobile ultrasound device.

Ultrasound assessments were performed using a Fujifilm SonoSite Edge II portable ultrasound machine equipped with a high-frequency linear transducer (13–6 MHz). Imaging was conducted in B-mode with the depth set to 15 cm, and the gain optimized to enhance visualization of the anterior pelvic ring structures. All assessments were performed in a standardized supine cadaver position, and dynamic evaluations included manual compression of the iliac wings to simulate closure of the symphyseal diastasis.

### Evaluation of the ultrasound images

**Fig. 1:**
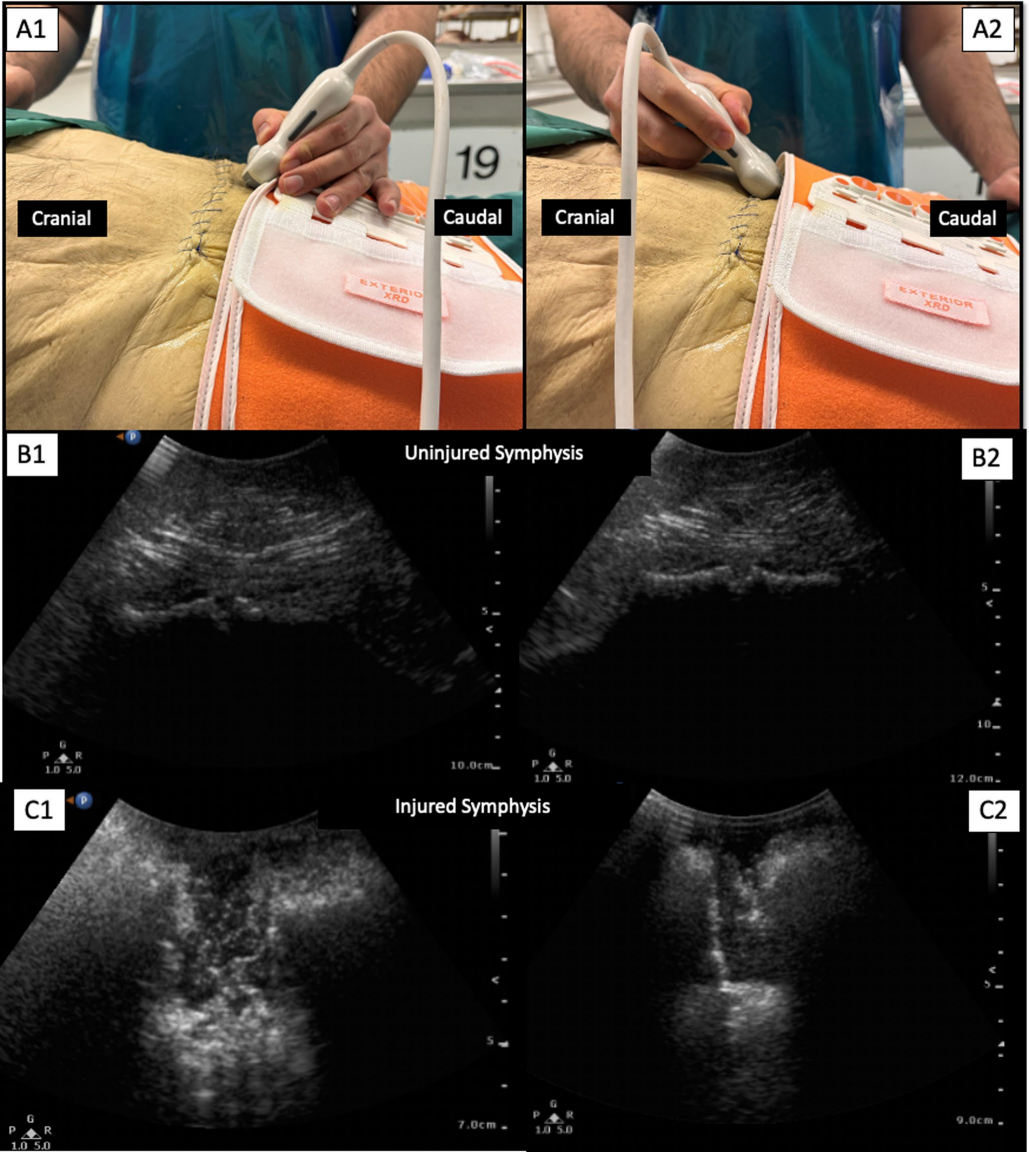
**A**: Clinical image of two different views on the symphysis. **B**: Ultrasound image of the uninjured symphysis (Control). **C**: Ultrasound image of the injured symphysis (here with diastasis of above2.5 cm)

**Fig. 2:**
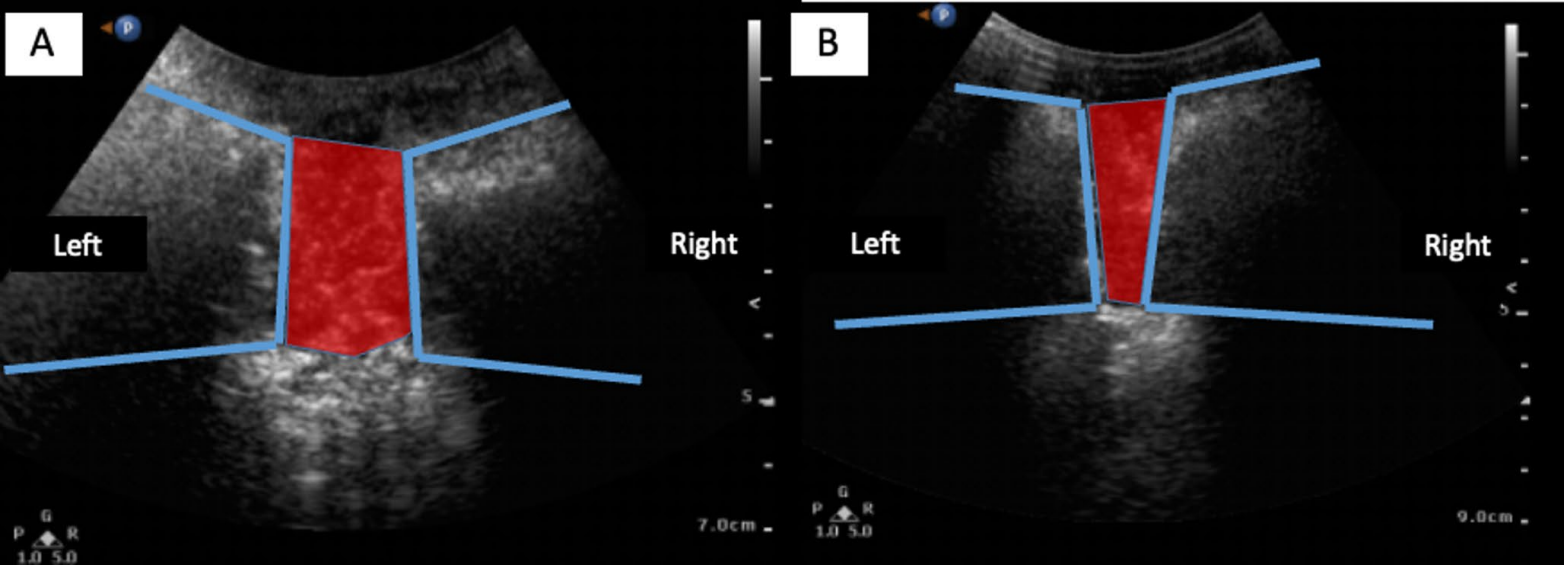
Schematics of the symphpysis (blue line) and the diastasis (red area). **A**: transducer tilted caudally. **B**: transducer tilted cranially

**Fig. 3:**
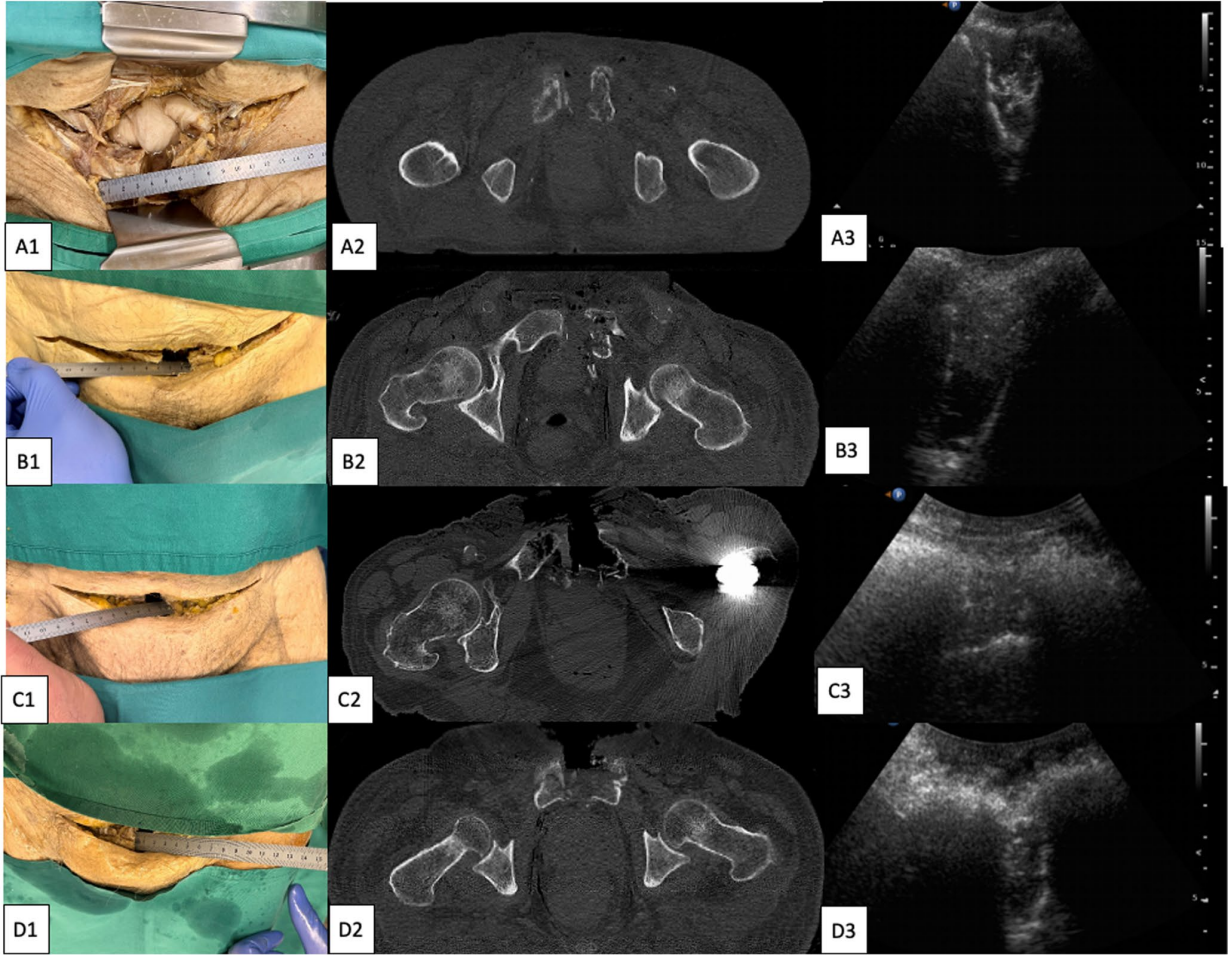
Each cadaver (**A**-**D**) with one exemplary diastasis with clinical measure, corresponding CT-scan and ultrasound image

**Fig. 4:**
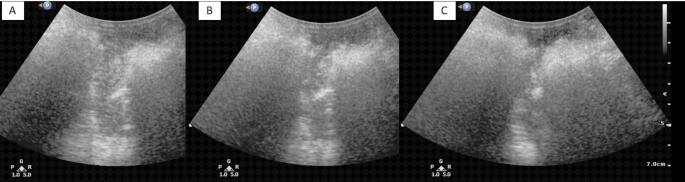
Dynamic assessment of the closure of the symphyseal diastasis. **A**: Symphyseal diastasis of 3.0 cm, **B**: Symphyseal diastasis of 2.0 cm; **C**: Closed symphysis

The cadaver were placed in a supine position and the transducer of the ultrasound was placed at the symphysis. Two different views were assessed: One with the transducer tilted cranially (Berk view), and the other with the transducer tilted caudally (Halva view). The clinical image and the corresponding ultrasound image are seen in Fig. 1. The bony structure of the anterior pelvic ring and the diastasis can be visualized in both views (Fig. 2). Each Diastasis was measured with a ruler. A CT scan served as a further gold standard control of the injury (Fig. 3). The dynamic assessment of the symphyseal diastasis included the continuous measurement of the injury with the ultrasound and the simultaneous manual closure of the symphysis in typical clinical manner (Fig. 4). The dynamic assessment was performed with one physician handling the ultrasound and assessing the symphysis and a second physician aiming for closed reduction by placing the hands on each side of the pelvis and compressing the pelvic ring.

### Blinded assessment

The participants are trauma surgeons with certain experience in the trauma bay at a level 1 trauma center. All participants were at least ATLS providers. The participants were blinded to the symphyseal diastasis. After a short training on the handling of the ultrasound device and the views that are being assessed, the participants performed a measurement of the symphysis. They were asked whether the symphysis was widened and whether they would apply a pelvic binder. Their measurements and their responses were documented.

### Statistics

Sensitivity and specificity analyses were performed with true positive defined as the following: pelvic binder would be closed following the ultrasound measure and the diastasis was truly injured. Sensitivity, specificity, positive predictive and negative predictive values were calculated and presented with 95% confidence intervals (CI). Descriptive statistics were calculated for sensitivity, specificity, positive predictive value (PPV), and negative predictive value (NPV) across four assessment groups: control, diastasis < 2.5 cm, diastasis > 2.5 cm, and dynamic closure. The diastasis values were based on the Young and Burgess classification [11]. Group comparisons of diagnostic performance were conducted using Fisher’s exact test to account for the small sample size (*n* = 11 assessments per group). A p-value < 0.05 was considered statistically significant. All analyses were performed using R.

## Results

A total of 11 trauma surgeons participated in this study. Of these 9 were ATLS providers, and 2 were ATLS instructors. All had at least 2 years hands on experience in treating trauma patients in a trauma bay setting at a level one trauma center and the initial assessment of severely injured patients.

Across all assessments, the sensitivity of ultrasound to detect symphyseal diastasis varied depending on the degree of injury. In the control group, sensitivity was 0.73 (95% CI: 0.39–0.94), while the sensitivity dropped significantly in the < 2.5 cm group to 0.45 (95% CI: 0.17–0.77). In contrast, sensitivity improved to 0.82 (95% CI: 0.48–0.98) in the > 2.5 cm group, and reached the highest value during dynamic closure testing, at 0.91 (95% CI: 0.59–1.00).

### Specificity

Remained consistently high across most groups, with the control, > 2.5 cm, and dynamic closure groups all showing a value of 1.00 (95% CI: 0.72–1.00). The only reduction was observed in the < 2.5 cm group, which had a specificity of 0.73 (95% CI: 0.39–0.94).

Analysis of positive predictive value (PPV) and negative predictive value (NPV) followed similar trends. PPV was 1.00 in all groups except < 2.5 cm, where it was 0.62 (95% CI: 0.24–0.91). NPV ranged from 0.57 in the < 2.5 cm group to 0.92 in the dynamic closure group (Table 1).

**Table 1:** Sensitivity analyses of ultrasound guided assessment pf symphyseal diastasis (95% Confidence interval)

	Control	Below 2.5cm	Above 2.5cm	Dynamic closure
Sensitivity	0.73 (0.39, 0.94)	0.45 (0.17, 0.77)	0.82 (0.48, 0.98)	0.91 (0.59, 1.00)
Specificity	1.00 (0.72, 1.00)	0.73 (0.39, 0.94)	1.00 (0.72, 1.00)	1.00 (0.72, 1.00)
Postive predictive value	1.00 (0.63, 1.00)	0.62 (0.24, 0.91)	1.00 (0.66, 1.00)	1.00 (0.69, 1.00)
Negative predictive value	0.79 (0.49, 0.95)	0.57 (0.29, 0.82)	0.85 (0.55, 0.98)	0.92 (0.62, 1.00)

## Discussion

Pelvic ring injuries might result in devastating complications if not treated appropriately. The early assessment of pelvic ring injuries is crucial. The present study proposes a standardized tool for the early assessment of open book injuries and found the following main results:

- Ultrasound reliably detected larger symphyseal diastasis (> 2.5 cm) and performed best during dynamic closure, which simulates real-time pelvic manipulation (applying pelvis binder).
- The marked drop in sensitivity for < 2.5 cm diastasis (0.45) highlights a limitation of ultrasound in detecting subtle pelvic injuries.
- The dynamic assessment method appears to enhance diagnostic accuracy and may be particularly useful in the trauma bay setting.

Preclinical application of a pelvic belt has become the standard in cases of suspected pelvic injury or a corresponding trauma mechanism [1, 12, 13]. Preclinical reduction of open pelvic injuries can be achieved with the pelvic belt, since especially severe anterior-posterior compression injuries, “open book injuries”, can be associated with vascular bargain [14]. However, a great deal of experience is required in the strength with which a pelvic belt should be tightened in order to avoid under compression or over compression of the pelvis. It is also difficult to assess whether this is the case in the individual patient, especially in the prehospital phase of polytrauma care.

As most pelvic belts are applied during the pre-hospital treatment algorithm, this can lead to lacking necessary experience with the required force that needs to be applied from the treatment team in the trauma bay. Studies on the force required to reposition the pelvic with the help of the pelvic binder are significantly underrepresented in literature. In particular, young, inexperienced trauma trainees may have difficulty with this. The dynamic US of the symphysis could be a quick, reproducible, and easy-to-use adjunct to the application of the pelvic binder or to re-access its tightness.

One could argue that a dynamic examination of the symphysis is also possible using conventional radiology. However, this method has not yet become established in the acute phase of trauma bay care. One reason for this could be the high radiation exposure for the patient and treatment team. In severely injured patients, many specialist disciplines are treating the patient simultaneously to quickly address life-threatening A, B, C or D problems [1]. A conventional x-ray can severely disrupt and interrupt these processes. In addition, “runners” who are potentially not equipped with x-ray aprons have to leave the trauma bay at short notice. Furthermore, the x-ray machine itself, if it is a mobile device, represents a physical obstacle to care. In our level 1 trauma center, it is also the case that everyone moves away from the patient during x-rays in order to keep scattered radiation to a minimum for the staff. This leads to interruptions in ongoing processes even with a single x-ray image taken. In the case of a dynamic examination, this could even result in the patient receiving inadequate care due to interruptions. Since a trauma spiral is usually performed following the primary survey according to ATLS, critical radiation exposure can occur, especially in pediatric patients. Williamson et al. found that 43.5% of pelvic binders were not correctly positioned. Specifically, 39.7% were placed too high (above the greater trochanters), and 3.8% were too low [15]. Incorrect positioning mite make anatomical repositioning of pelvic fractures much more difficult. Another retrospective analysis by Naseem et al. showed that of 140 patients with pelvic binders, only 49.1% had satisfactory placement. These discrepancies may compromise the mechanical efficiency of the binder and the clinical outcome [16].

Incorrect placement of pelvic binders can reduce their effectiveness in stabilizing the pelvic ring and controlling hemorrhage. These findings underscore the need for improved education and training in both pre-hospital and in-hospital settings. Simulation training, refresher courses, and standardized protocols may improve the consistency and accuracy of pelvic binder application. Moreover, tools such as the presented dynamic US may support clinicians in achieving correct anatomical reductions of the symphysis.

E-FAST is a recognized adjunct in polytrauma care [17]. In addition to the diagnosis of the lungs and heart, the abdomen and pelvis can be examined for free fluid. In clinical practice, this is carried out in the order listed above. From the examination of the pelvis, the sonography transducer is already in the ideal position to examine the symphysis. By rotating the transducer caudally, the diagnosis of bleeding sources can be complimented with the pelvis accurately in a minimum of time. Our data showed regarding sensitivity: This trend suggests that the diagnostic performance of ultrasound increases with the degree of diastasis and when the joint is manipulated under dynamic conditions. Regarding specificity: These findings indicate that when ultrasound identified a negative case (no diastasis), it was highly reliable, especially in cases with more pronounced or dynamically tested injuries. The PPV showed that these values suggest that ultrasound is particularly strong in confirming the presence of diastasis, though its ability to confidently rule out injury diminishes in cases of subtle displacement. Overall, the data support the feasibility of using ultrasound for anterior pelvic ring assessment, with greatest diagnostic accuracy for larger or dynamically tested injuries.

It has been further shown that the pelvis binder application can lead to a concealment of pelvic ring instabilities [8]. In such cases, dynamic ultrasound imaging may be helpful when opening the pelvic binder to rule out occult symphysis injuries. (Reversed dynamic ultrasound).

### Strengths & limitations

Clear strengths of our study are the blinding and randomization to reduce biases. However, we are aware of certain limitations. A major limitation is that the study was a cadaver study which were chemically treated. This fact makes it difficult to compare to real patients, since swelling, hematoma and soft tissue injuries, which can indicate fractures, are not visible. Further there is no patient history present or sounds of pain / discomfort when testing the pelvis that could also give clous of the presents of a pelvis trauma. Lastly, the quality of the ultrasound strongly depends on the amount of water in the examining tissue. Since the cadavers are chemically treated, this could influence the imaging quality and therefore the results. Since ultrasound measurement of the symphysis is a new method that is not yet established in everyday clinical practice or in ATLS, we assume that all participants in the study are equally inexperienced with the measurement. This makes it difficult to assess inter-rater reliability or intra-rater reproducibility and needs to be verified in further studies.

While the overall diagnostic performance of the ultrasound technique demonstrated high sensitivity and specificity, subgroup analysis revealed a notably low negative predictive value (NPV) of 0.57 for injuries measuring less than 2.5 cm. This finding indicates a substantial risk of false negatives in this subset, which raises concerns about the potential for missed diagnoses. Clinically, this underscores the importance of interpreting a negative ultrasound result with caution, particularly when evaluating for smaller or more subtle pelvic injuries. As such, ultrasound should be used strictly as an adjunct to—not a replacement for—comprehensive trauma evaluation protocols, which may include CT imaging and clinical correlation, especially when clinical suspicion remains high. Due to the retrospective nature of the study and sample size constraints, additional subgroup analyses or experiments to further refine the precision of NPV were not feasible. This limitation highlights the need for future studies to explore strategies for improving diagnostic accuracy in smaller lesions and should be considered when applying these findings in clinical practice.

The integration of the PRIME assessment into the E-FAST protocol offers promising diagnostic value; however, its feasibility within the ATLS framework requires careful consideration. The PRIME maneuver can be performed rapidly (typically under 30 s) by a trained sonographer or trauma team member during the “D” or “E” phases of the primary survey. Importantly, it does not require additional equipment beyond the ultrasound probe already in use for E-FAST, and the dynamic manipulation can be synchronized with other clinical tasks. Nevertheless, in high-acuity polytrauma cases, workflow congestion and personnel limitations may restrict its immediate implementation. Therefore, while we believe PRIME is operationally feasible in most well-coordinated trauma settings, its integration should be tailored to institutional protocols and available resources. We recommend that PRIME be performed by the clinician responsible for the E-FAST, ideally before definitive imaging, and only when it does not compromise the primary resuscitation effort. Future studies should assess the real-world impact of PRIME on trauma team efficiency and patient outcomes to validate its role further.

One important limitation of this study is the use of formalin-fixed cadavers, which introduces key differences compared to living tissue. Formalin fixation alters tissue elasticity, causes dehydration, and increases stiffness, all of which affect ultrasound propagation and image quality. These changes may influence the appearance of anatomical landmarks and fluid-tissue interfaces, potentially impacting both the sensitivity and specificity observed in this study. As a result, the diagnostic performance reported here may not fully translate to clinical practice, where tissue characteristics and physiological conditions differ significantly. While cadaveric models are valuable for the standardized evaluation of novel techniques such as PRIME, further validation in live trauma patients is necessary to confirm clinical accuracy and utility.

## Conclusion

Ultrasound reliably detected larger symphyseal diastasis and performed best during dynamic closure, which simulates real-time pelvic manipulation such as applying a pelvis binder. Especially the dynamic assessment method appears to enhance diagnostic accuracy and may be particularly useful in the trauma bay setting. Further prospective, randomized, blinded studies on actual patients are needed to generate more knowledge about the possibilities of PRIME ultrasound.

## Data Availability

No datasets were generated or analysed during the current study.
